# Septic arthritis caused by *Brucella melitensis* in urban Shenzhen, China: a case report

**DOI:** 10.1186/1752-1947-8-367

**Published:** 2014-11-14

**Authors:** Tak Man Wong, Nan Lou, Wentao Jin, Felix Leung, Michael To, Frankie Leung

**Affiliations:** 1 Department of Orthopedics and Traumatology, The University of Hong Kong, Queen Mary Hospital, 102, Pokfulam Road, Hong Kong, China; 2 Shenzhen Key Laboratory for Innovative Technology in Orthopaedic Trauma, The University of Hong Kong-Shenzhen Hospital, 1 Haiyuan 1st Road, Futian District, 518053 Shenzhen, China; 3 Department of Orthopedics and Traumatology, The University of Hong Kong-Shenzhen Hospital, 1 Haiyuan 1st Road, Futian District, 518053 Shenzhen, China

**Keywords:** Brucellosis, *Brucella* species, Septic arthritis, Hip arthroscopy, Urban area

## Abstract

**Introduction:**

Brucellosis is a systemic infectious disease which is still a challenging medical problem in rural areas such as northern China. It rarely occurs in urban areas such as Shenzhen in southern China. Osteoarticular involvements are frequently seen in brucellosis, and rarely is arthritis the only clinical presentation. We report a case of hip septic arthritis caused by *Brucella melitensis* in an urban area of Shenzhen, China.

**Case presentation:**

A 29-year-old Chinese woman, Han ethnical group presented to our hospital with left hip pain persisting for one month. She had a history of contact with goats one month before admission. Her clinical examination showed marked tenderness and limited movement of her left hip. Further imaging showed effusion of her left hip joint. Inflammatory markers including erythrocyte sedimentation rate (ESR) and c-reactive protein (CRP) were raised. Our clinical diagnosis was septic arthritis of the left hip. A left hip arthroscopy was performed and the culture was positive for *Brucella melitensis*. She returned to normal activity after completing a standard antibiotic regimen, including gentamicin at 120mg daily for 2 weeks, doxycycline at 100mg daily and rifampicin at 450mg for a total of 12 weeks.

**Conclusions:**

Brucellosis is endemic in some rural areas of China, but rare in urban areas such as Shenzhen in southern China. However, more cases will be expected in urban areas due to increasing migration within China. Physicians should consider brucellosis as one of the differential diagnosis of arthritis. Early surgical intervention is recommended to prevent further joint destruction.

## Introduction

Brucellosis is a highly contagious zoonosis caused by *Brucella. Brucella* species are small, gram-negative non spore-forming rods or coccobacilli. Many animals are susceptible to infection by *Brucella* species, such as goats, cows, pigs and sometimes canines. It spreads firstly between animals and then from animals to humans. *Brucella* infection can occur via genital mucosa, inhalation, digestion and even via injured skin. Human infection is usually through the inspiration of aerosols containing *Brucella*, ingestion of unpasteurized milk or undercooked meat from infected animals, or close contact with their secretions, baby animals, milk and excrement. Transmission from human to human has been rarely reported. In China, brucellosis is still endemic in rural areas, but is rare in urban areas such as Shenzhen in southern China. Although vaccines have been used to prevent human brucellosis [1], the incident rate of brucellosis is still increasing in recent years. In 2010, reported cases of brucellosis numbered around 33,000 [2].

The clinical features of human brucellosis are undulant fevers, sweating, migratory arthralgia, myalgia and hepatosplenomegaly, and some of these may take a chronic form. Since complications are common and symptoms may be atypical with various presentations, brucellosis is easily misdiagnosed and thus treatment is delayed. Monoarthritis of knee joints caused by *Brucella* species have been reported [3], but rarely hip joints. We describe the case of a woman who presented with left hip pain due to septic arthritis caused by *Brucella melitensis* in Shenzhen, China.

## Case presentation

**Figure 1:**
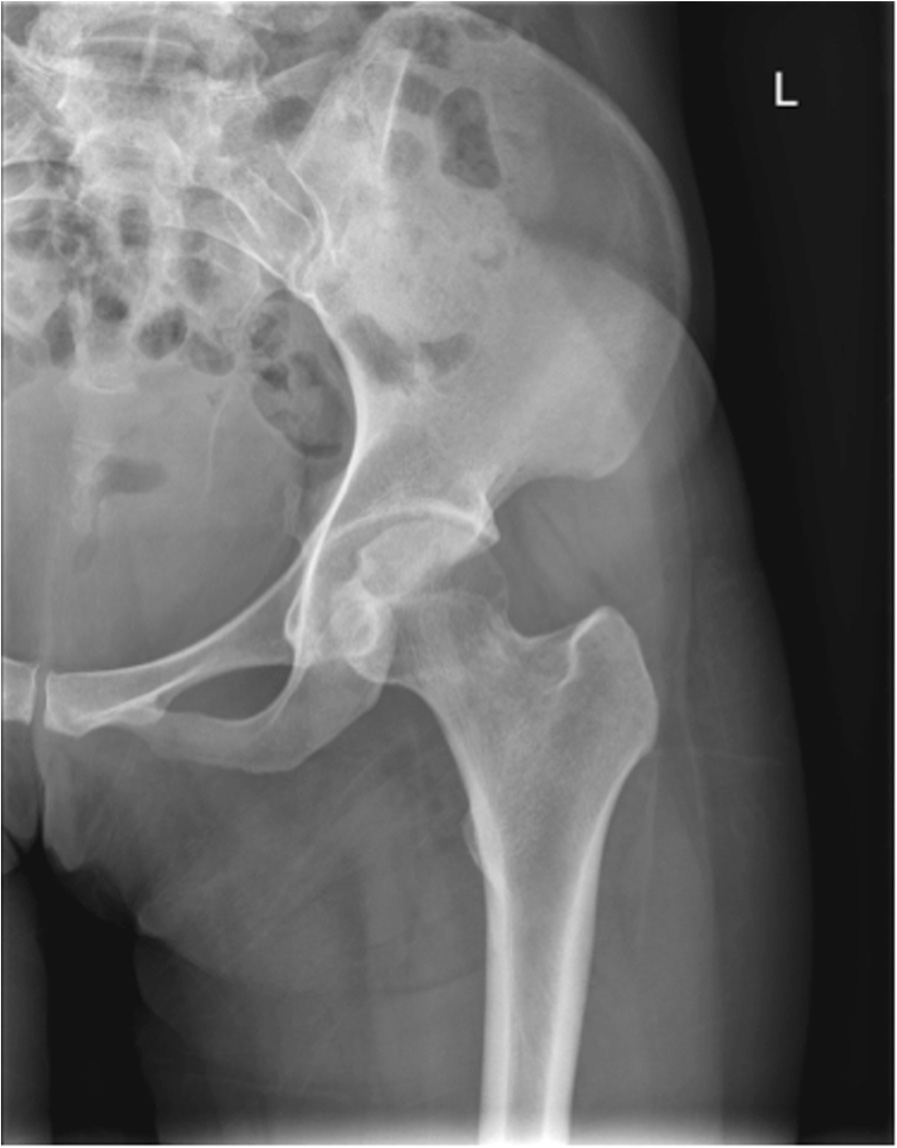
Normal X-ray left hip - Anteroposterior view.

**Figure 2:**
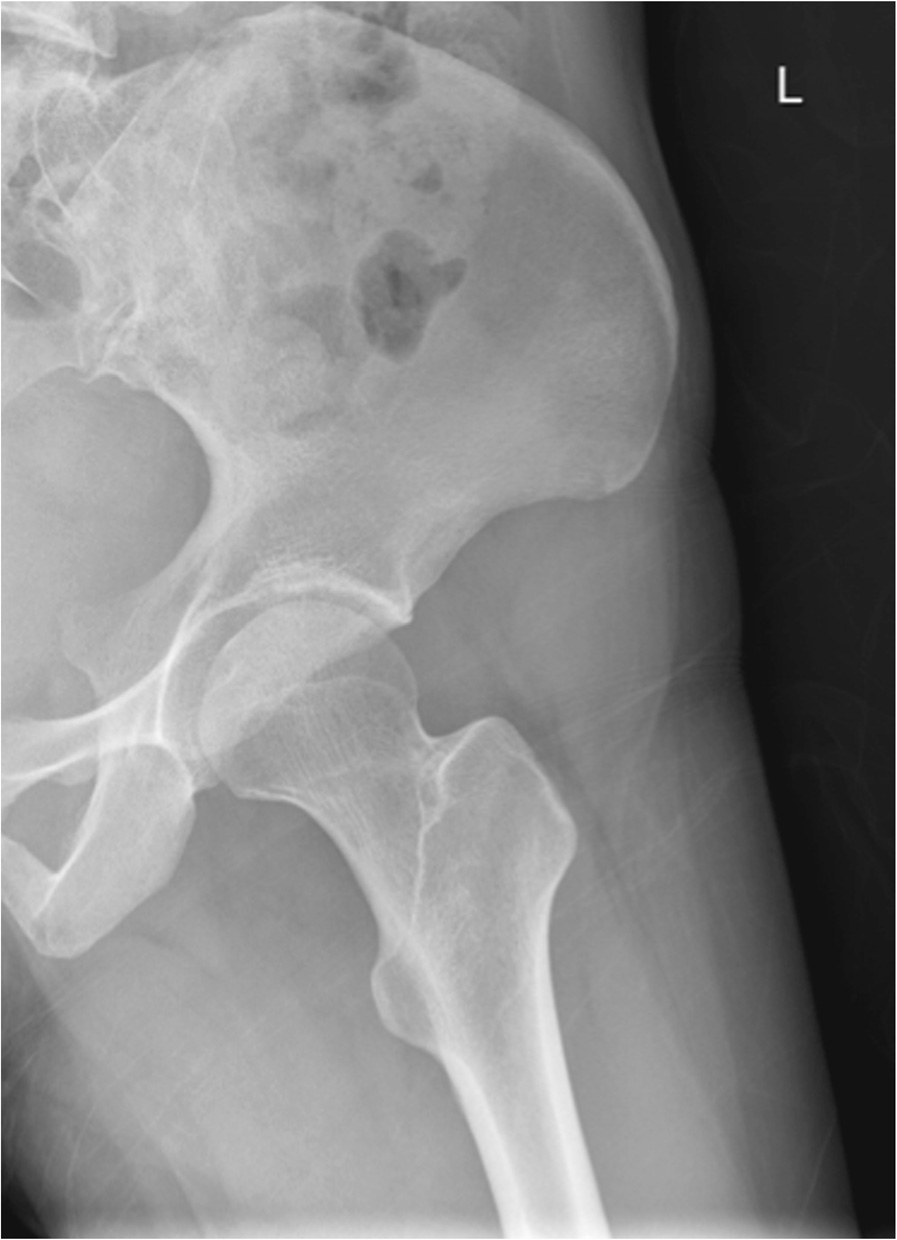
Normal X-ray left hip - Lateral view.

**Figure 3:**
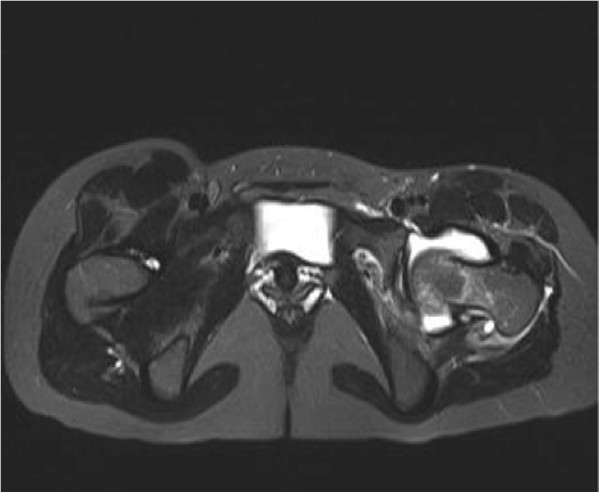
T2-weighted magnetic resonance imaging transverse plane of pelvis showed marked effusion over left hip joint.

**Figure 4:**
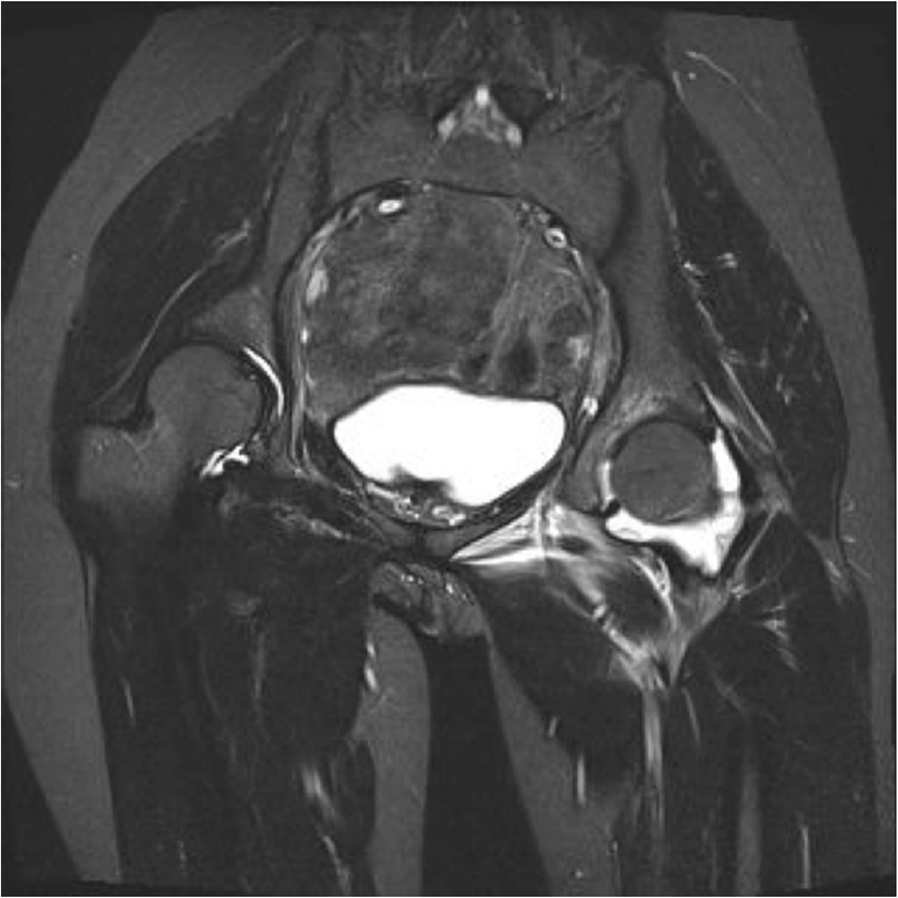
T2-weighted magnetic resonance imaging coronal plane of pelvis showed marked effusion over left hip joint.

A 29-year-old Chinese woman living in Shenzhen, China, presented to our hospital with pain and limited movement in her left hip for one month previous. She had no relevant medical history, with no history of injury or alcohol or steroid use. She visited other hospitals and non-steroidal anti-inflammatory drugs were given, however there had been no improvement. According to her history, she had direct contact with goats at her mother’s house one month previous. Her mother is a shepherd in rural area in Henan province in northern China and she fed some baby lambs during the visit. On admission, she was afebrile and all vital signs were stable. Her left hip was positioned at a 45-degree flexion and 20 degree external rotation. There was tenderness around the left groin region. Left hip rolling and axial loading tests showed marked tenderness over her left hip. Active and passive movements of her left hip were limited because of significant pain. The rest of her physical examination was unremarkable. Blood tests revealed her white cell counts, liver and renal parameters to be within normal limits and her rheumatoid factor was negative. Her erythrocyte sedimentation rate (ESR) and C-reactive protein (CRP) were elevated, as shown in Table 1. An initial X-ray of her pelvis was unremarkable (Figures 1 and 2). A magnetic resonance imaging (MRI) scan of her left hip (Figures 3 and 4) showed marked joint effusion and synovitis. Our clinical diagnosis was that of left hip septic arthritis.

**Table 1:** Biochemical results

**Time**	**Preoperative**	**Immediate Postoperative**	**Postoperative 1 month**	**Postoperative 3 months**
**Results**				
White blood cell count (x10^9^/L)	Normal	Normal	Normal	Normal
Erythrocyte sedimentation rate (mm/hr) (normal<10)	69	77	30	8
C-reactive protein (mg/L) (normal<5)	31.9	37.3	3.0	1.5

In view of her significant symptoms, a left hip arthroscopy and synovectomy was performed on the day after admission. Intraoperative findings showed turbid joint fluid together with significant synovitis. A joint fluid culture and synovial tissue culture showed the presence of *Brucella melitensis*. A standard tube agglutination test was positive at a titre of 1/200.

A standard antibiotic regimen was initiated after operation, including gentamicin at 120mg daily for 2 weeks, doxycycline at 100mg daily and rifampicin at 450mg for a total of 12 weeks.

**Figure 5:**
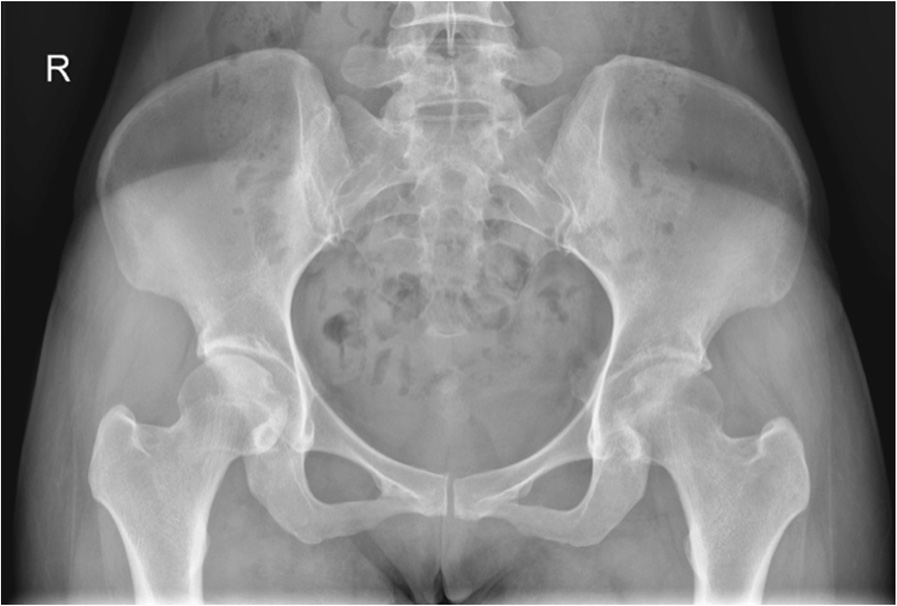
X-ray of her pelvis three months after surgery – Anteroposterior view showed mild osteopenic change over the left femoral head but joint space was preserved.

**Figure 6:**
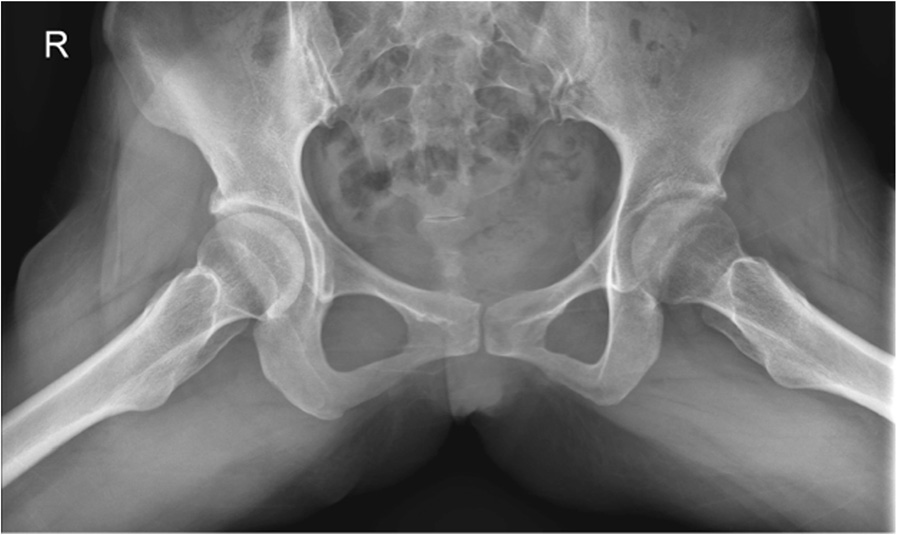
X-ray of her pelvis three months after surgery - Frog view showed mild osteopenic change over the left femoral head but joint space was preserved.

Serial blood tests showed ESR and CRP were improved after treatment (Table 1) and she returned to normal activity two months after treatment was initiated. An X-ray of her pelvis showed a mild osteopenic change over the left femoral head but joint space had been preserved (Figures 5 and 6).

## Discussion

Brucellosis is still an endemic infection and a challenging health problem in many parts of the world, especially in the Near and Middle East, Latin America, Africa and China. In China, brucellosis is more frequent in northern China in pasturing areas such as Inner Mongolia, Hebei, Henan, Shangdong and Tibet. It seldom occurs in the southern part of China, however, in recent years human brucellosis has been spreading quickly from rural to urban areas and there has been a sharp increase in reported cases in southern China. There were more than 60 cases reported in the past 5 years in Guangzhou (South China) [4]. One possibility is that infected animals and raw products are transported across the whole country. This may put ordinarily low-risk persons at much higher risk when they consume or handle infected raw products.

In our case, contact with infected goat in Henan was the most likely source of infection. This might be another reason accounting for the increase in cases of human brucellosis in urban areas, as the traffic between rural and urban areas within China is increasing.

Brucellosis usually affects young or middle-aged adults, with low incidence rates in children and the elderly [5]. The incubation period is around 2 to 4 weeks, before symptom and signs appear. Usually the symptoms of brucellosis are non-specific, such as joint pain, fatigue, sweating, fever and gastrointestinal problems. Pourbager *et al.* reviewed that osteoarticular complaints were the most common symptoms [6]. They may present as arthritis, spondylitis, bursitis, tenosynovitis and osteomyelitis. Sacroiliac joint is the most common site of involvement [7], followed by knee or ankle joints [8]. Monoarthritis of the hip in children [9] and even prosthetic hip infections [10] caused by *Brucella*e have been previously reported, but rarely in healthy young adults.

The diagnosis of brucellosis is based on history, clinical examination and biochemical results. Several serological tests have been developed to measure the antibodies against *Brucella*. The standard tube agglutinin test is widely used, titres of ≥160 are considered positive, although using 320 as cutoff may be more specific in endemic areas [11]. The gold standard for confirmation of brucellosis is to perform an arthrocentesis to culture the organism, using the Bac-Tec™ blood culture system (Becton Dickinson Diagnostix Instrument Systems, Towson, Md., United States of America) from multiple samples. Unlike septic arthritis caused by other organisms, examination of the synovial fluid should show a leukocyte count of less than 50,000cells/mm^3^, with a predominance of lymphomononuclear cells. Ayaşlıoğlu *et al.* reported a synovial leukocyte count of 8000 cells/mm^3^ in one patient with septic brucellar monoarthritis [12], whereas Andonopoulos *et al.* also reported synovial counts ranging from 4460 cells/mm^3^ to 8800 cells/mm^3^ in five patients with the same disease [13].

Treatment of brucellosis should involve antibiotics and surgeries if necessary. The standard antibiotic regimen is doxycycline combined with streptomycin or rifampicin for 6 week**s**[14]. Skalsky *et al.* suggested triple regimens including an aminoglycoside [15] and offers an advantage over doxycycline with an aminoglycoside or rifampicin. Therefore, triple regimens were used in our case. Single agent treatment regimens are not recommended for osteoarticular involvement of brucellosis because of the high relapse rate. In our case, in view of significant symptoms, together with raised ESR and CRP, we performed a therapeutic hip arthroscopy followed by standard antibiotic regimens and nil complication was reported. To the best of our knowledge, this was the first case of *Brucella melitensis* septic arthritis treated by hip arthroscopy reported in the literature.

## Conclusions

Brucellosis is still a challenging medical problem in China. Due to increasing traffic between rural and urban areas, more cases in urban areas are to be expected. Physicians should consider brucellosis as one of the differential diagnosis of arthritis, irrespective of whether they are in endemic or non-endemic areas. Detailed travel and contact histories of patients are essential in order to establish an early diagnosis. In cases of osteoarticular involvement, early surgical interventions together with antibiotics are recommended to prevent further destruction of the joints.

## Consent

Written informed consent was obtained from the patient for publication of this case report and accompanying images. A copy of the written consent is available for review by the Editor-in-Chief of this journal.

## Abbreviations

CRP: C-reactive protein; ESR: Erythrocyte sedimentation rate.

## Competing interests

The authors declare that they have no competing interests.

## Authors’ contributions

NL and WJ were involved in the writing of the report. TMW, MT and Fr-L participated in writing and editing the manuscript. Fe-L participated in the discussion part and literature review. All authors read and approved the final version of the manuscript.
